# Applying GIS and Machine Learning Methods to Twitter Data for Multiscale Surveillance of Influenza

**DOI:** 10.1371/journal.pone.0157734

**Published:** 2016-07-25

**Authors:** Chris Allen, Ming-Hsiang Tsou, Anoshe Aslam, Anna Nagel, Jean-Mark Gawron

**Affiliations:** 1 Department of Geography, San Diego State University, San Diego, California, United States of America; 2 Graduate School of Public Health, San Diego State University, San Diego, California, United States of America; 3 Department of Linguistics, San Diego State University, San Diego, California, United States of America; Qom University, ISLAMIC REPUBLIC OF IRAN

## Abstract

Traditional methods for monitoring influenza are haphazard and lack fine-grained details regarding the spatial and temporal dynamics of outbreaks. Twitter gives researchers and public health officials an opportunity to examine the spread of influenza in real-time and at multiple geographical scales. In this paper, we introduce an improved framework for monitoring influenza outbreaks using the social media platform Twitter. Relying upon techniques from geographic information science (GIS) and data mining, Twitter messages were collected, filtered, and analyzed for the thirty most populated cities in the United States during the 2013–2014 flu season. The results of this procedure are compared with national, regional, and local flu outbreak reports, revealing a statistically significant correlation between the two data sources. The main contribution of this paper is to introduce a comprehensive data mining process that enhances previous attempts to accurately identify tweets related to influenza. Additionally, geographical information systems allow us to target, filter, and normalize Twitter messages.

## Introduction

Public health scholars have long studied the ways in which disease outbreaks can be monitored. Accurate information about the intensity and geographic distribution of illnesses can allow officials to allocate resources effectively and respond appropriately in order to combat these health threats. However, surveillance of influenza has traditionally been problematic due to uneven and haphazard reporting, as well as the time and cost overhead associated with gathering this information.

This paper introduces a geo-targeted data mining approach to influenza surveillance that relies upon data from the social media platform Twitter. Messages from Twitter are gathered and analyzed using geographic information system (GIS) methods such as spatial filtering, population normalization, and multi-scale analysis. Additionally, this work also turns to machine learning techniques to improve the filtering process in order to better distinguish tweets that appear to describe real-world cases of influenza from those that do not. For this purpose, a support vector machine (SVM) classifier was trained using manually tagged data collected from the 2012–2013 flu season. Comparing our results for the 2013–2014 flu season with official reports of influenza-like illness (ILI) on national, regional, and local levels demonstrates that the two signals are closely correlated, suggesting that Twitter holds great potential for monitoring influenza outbreaks.

## Background and Literature Review

This study builds on the flurry of recent research activity seeking to understand the dynamics of disease outbreak by analyzing Internet and social media data. Though remarkable progress has been made in the past few years, there remain significant obstacles facing researchers wanting to use these new sources of data to inform public health decisions.

Many researchers have turned to Google Flu Trends (GFT) for user-generated data on influenza. This site publishes information on flu-related web searches that can be used to monitor changes in Internet activity over time and space. Dukic et al. [1] modified the classical particle learning epidemiology model using GFT data to develop a method for monitoring the spread of flu. More recently, Dugas et al. [2] have relied upon historical outbreak data along with patterns in Flu Trends to create a forecast model that allows the team to predict flu cases one week in advance with reasonable precision. In the past few years, however, many have grown skeptical of the accuracy of Google Flu Trends. Olson et. al. [3], for example, argue that GFT predictions are not accurately tuned to yearly variations in flu patterns. Similarly, Declan Butler has commented in *Nature* [4] that Google’s significant miscalculation in predicting the intensity of flu outbreaks for the 2013 season casts serious doubt on the reliability of Flu Trends data to monitor illness.

In the past few years, the micro-blogging service Twitter has provided researchers with a rich new source of data, allowing for detailed examinations of complex diffusion processes, human behaviors, and collective attitudes around the world [5–7]. Scholars from divergent fields have similarly turned to Twitter to collect, filter, and analyze messages (called tweets) in order to gain new insights into the spread of flu. Nagel et al. [8], for instance, have developed a methodology for collecting and filtering tweets that demonstrates a high correlation with local and national reports of ILI cases. Other researchers have delved deeper into text analysis to more accurately remove tweets that are unrelated to actual cases of influenza. Notably, Lamb et al. [9] used manually-defined word feature classes to filter out tweets that are did not appear to reflect personal infection.

Though these efforts are valuable contributions to the fields of public health and Big Data analysis, this paper suggests that a greater reliance on geographic information system (GIS) and machine learning methods can shed new light on the role these exciting new data sources (particularly Twitter) can play in studying disease outbreak.

## Data Collection

The data collection procedure for this research was based upon the Visualizing Information Space in Ontological Networks (VISION) framework developed by Tsou, et. al. [10–11] to examine the interrelationships between cyberspace message, space, and time. Unlike most previous applications of the VISION framework, this study focuses exclusively on data collected from Twitter due to the real-time and dynamic nature of this platform.

In the past few years Twitter has emerged as one of the leading social media platforms, boasting more than 140 million active users. Each day, Twitter users produce millions of tweets (messages of 140 characters or less), which can be collected through official Twitter APIs. To assist with our data collection process, our research group has developed search tools which query the Twitter Search API based on spatial constraints and keyword filters. To allow researchers within our group to visually explore this data, a web map was developed that displays the search locations related to each keyword, as well as the intensity for that keyword. Using this interface, it is also possible to download the Twitter data in Excel form, which allows for a more detailed analysis of the various attributes associated with each tweet, such as the time it was published, the user’s location, the GPS coordinates of the tweet, and any URLs or hashtags contained within the message (see S3 File).

This study builds on the work of Nagel et al. [8] by using keywords that have been identified as effective indicators of influenza outbreak. The Twitter search tool collected tweets by the keywords “flu” and “influenza.” Tweets were collected from 31 major cities in the United States: Atlanta, GA; Austin, TX; Baltimore, MD; Boston, MA; Chicago, IL; Cleveland, OH; Columbus, OH; Dallas, TX; Denver, CO; Detroit, MI; El Paso, TX; Fort Worth, TX; Houston, TX; Indianapolis, IN; Jacksonville, FL; Los Angeles, CA; Memphis, TN; Milwaukee, WI; Nashville, TN; New York, NY; Oklahoma City, OK; Philadelphia, PA; Phoenix, AZ; Portland, OR; San Diego, CA; Seattle, WA; and Washington D.C. Unlike Nagel et. al., this research uses variable search radiuses for each of these cities, which were determined by the researchers.

## Methods

For this study, geographic information system (GIS) methods were relied upon for data collection and normalization. To filter out noise from the dataset, a machine learning procedure was adopted that allowed our group to better distinguish tweets that appeared to indicate a real-world instance of influenza from those that did not.

In order to target specific locations for data collection, this project took advantage of the spatial filtering methods provided by the Twitter Search Application Programming Interface (API) (see S3 File). Most research focusing on Twitter data relies upon the Streaming API, which allows users to retrieve GPS-tagged tweets within a specified bounding box. However, the main disadvantage of this method is that the Streaming API only gives access to a one percent sampling of all tweets. By polling the Search API continually, we can access a much larger dataset for specific geographic areas, which allows for detailed analysis of the data at municipal, regional, and national scales.

Normalization of population is essential to understanding quantitative geographic data, and for this study a novel approach was taken to carry out this task. The Twitter Search API requires that geo-targeted searches specify a latitude/longitude pair as well as a radius, which can essentially be thought of as a point buffer. Each of these city point buffers was joined with census tract centroids to determine which tracts should be included in our population calculations. Using the fine-grained census data allows us to gain a more accurate estimation of population, which greatly improves our ability to accurately normalize tweet counts for individual cities.

With respect to noise filtering, Nagel et al. [8] have shown that excluding retweets and tweets containing URL links produces a much higher correlation coefficient with ground-truth data, and we have borrowed that strategy in this study. However, to further filter our data, we have developed a machine learning classification procedure. The goal of this procedure was to identify tweets that do not appear to indicate real-world cases of influenza so that they can be omitted from the statistical analysis. The following are example tweets containing the keyword “flu” and the determination of their validity that we hoped to accomplish with the classification task:

- *Flu medicine kicked in*… *Time for bed* → Valid
- *I gotta get over this flu*!! → Valid
- *Who gets the stomach flu the day before class starts*? → Invalid
- *I'm getting the flu shot today*. *#scared* → Invalid
- *Arm is killing from flu jab* → Invalid
- *this flu feels like death* → Valid

As these examples show, in many cases, one can succeed at classifying tweets by recognizing one positive indicator (e.g., “get over”, “medicine”) or one negative indicator (e.g., “shot”, “jab”). This property strongly suggested to our team that using a linear learning algorithm would be an effective strategy.

In machine learning terms, the problem of assigning the labels “no” or “yes” to a tweet is a simple binary classification task. The features relevant to classification are words (such as “medicine” or “bed”) and n-grams (such as “stomach flu”, “flu shot”). The learning algorithm is given a training set of tweets represented as features with numerical values and labeled with their classes. With a linear classifier, the output of the learning algorithm is simply a set of feature weights; unseen examples can be classified using a linear combination of the weighted features. The primary advantage of using a linear classifier is that learning is cheap and scalable. Additional positive and negative indicators can be learned with more labeled data. Furthermore, the problem of what to do when indicators conflict is solved automatically by the feature weights. For instance, consider a tweet that contains both the word “stomach” and the word “medicine.” The learner classifies such tweets based on the weighting that was optimal in correctly classifying the training data. The particular kind of linear learner used here is called a support vector machine (SVM) [12].

In this context, an SVM was used to classify tweets that appeared to be indicators of real-world cases of influenza and those messages that appeared to be irrelevant to actual illness (see above examples). To train the SVM, 1,500 randomly sampled tweets from the 2012–2013 season containing the keyword “flu” were used to train the SVM classifier. Each of these tweets was manually inspected and classified as valid or invalid according to the likelihood that the message indicated an actual case of influenza. In order to assign numerical values to unigram, bi-gram, and tri-gram features, we use the term frequency—inverse document frequency (TF-IDF) scores for each word or word-pair. TF-IDF is a measure of the statistical significance of each term in a message that also accounts for the prevalence of that term in the overall data set. Essentially, this score assigns a higher weight to words that are more important to a specific message in comparison the entire set of messages [13]. It should be noted that using statistical transformations such as TF-IDF allows the researcher to avoid the task of manually identifying specific keywords indicating influenza; rather, the classifier is able to automatically recognize important keywords based on patterns in the manually classified training set. Consequently, applying this procedure to other illnesses (for example, Ebola) would simply require a new set of manually classified tweets with which to train the classifier.

To evaluate the classification model, we used standard machine learning measures of quality: *recall*, *precision* and the resulting *F1 score*. Using 1,000 randomly sampled tweets as a test set, we determined that the classifier has a precision score of 0.671, a recall score of 0.949, resulting in an F1 score of 0.786. This essentially means that the model was able to classify most valid tweets correctly (indicated by the high recall score), but as the precision score indicates, it occasionally incorrectly categorizes invalid tweets as valid.

## Results

The results demonstrate that this new procedure provides significant advantages over previous studies when comparing tweet rates to local, regional, and national ILI reports. These outcomes are summarized in Table 1, which shows the Pearson coefficient between each city’s tweet rate and both the regional and national ILI, as well as the coefficients between the tweet rate and local ILI, for cities where this data was available. Note that local ILI data is displayed from both emergency providers and sentinel providers. Although Aslam et al. [14] have shown that emergency reports produce a stronger signal, data from these sources are difficult to obtain, so sentinel reports are also included.

**Table 1:** Summary of correlations between tweet rates and ILI rates (local, regional, and national).

CITY	CORR. WITH LOCAL EMERGENCY ILI	CORR. WITH LOCAL SENTINEL ILI	CORR. WITH REGIONAL ILI	CORR. WITH NATIONAL ILI
Atlanta	NA	NA	0.657	0.679
Austin	NA	NA	0.919	0.830
Baltimore	NA	NA	0.031	-0.116
Boston	0.804*	0.105	0.395	0.433
Chicago	0.804*	0.636	0.771	0.782
Cleveland	0.784*	0.605	0.819	0.822
Columbus	0.877*	-0.235	0.771	0.776
Dallas	NA	NA	0.702	0.797
Denver	NA	0.690	0.599	0.589
Detroit	NA	0.757	0.846	0.878
El Paso	NA	NA	0.422	0.563
Fort Worth	NA	0.855	0.659	0.734
Houston	NA	NA	0.845	0.663
Indianapolis	NA	NA	0.750	0.777
Jacksonville	NA	NA	0.787	0.778
Los Angeles	NA	NA	0.793	0.690
Memphis	NA	NA	0.850	0.854
Milwaukee	NA	NA	0.761	0.779
Nashville	NA	0.827	0.869	0.875
New Orleans	NA	NA	0.858	0.886
New York	NA	0.555	0.630	0.639
Oklahoma City	NA	NA	0.463	0.658
Philadelphia	NA	NA	0.718	0.624
Phoenix	NA	NA	0.820	0.685
Portland	NA	NA	0.837	0.725
San Antonio	NA	NA	0.824	0.809
San Diego	0.916*	0.693	0.750	0.626
San Francisco	NA	NA	0.707	0.616
San Jose	NA	NA	0.715	0.653
Seattle	NA	0.830	0.807	0.665
Washington DC	NA	NA	0.756	0.578

* Emergency ILI reports were incomplete and thus the correlation only compares tweet rates with available ILI data. Boston is missing weeks 36–46, 48, 50, and 6–10. Chicago is missing weeks 36–40 and 6–10. Cleveland is missing 36–39 and 4–10. Columbus is missing weeks 36–39 and 6–10. San Diego is missing week 6–10.

**Fig 1:**
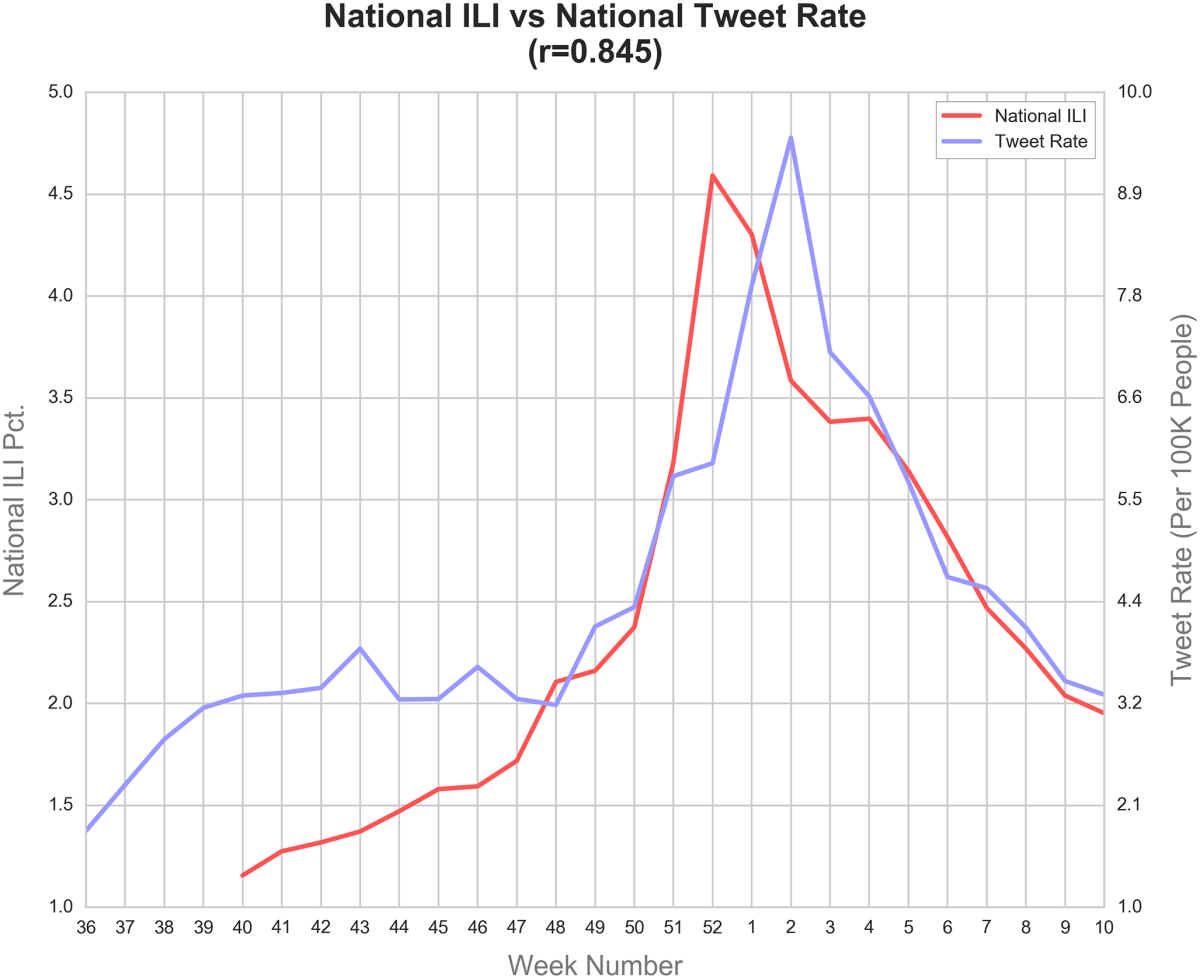
National ILI compared to the aggregated tweet rates for all study cities.

In order to understand how flu tweets are related to national ILI percentages, we aggregated data for each city to formulate a national tweet rate. As shown in Fig 1, comparing the national ILI to the aggregated tweet rate reveals that the two numbers are highly correlated (r = 0.845). Though this national comparison ignores possible spatial variability in flu outbreaks, it nonetheless serves as a good baseline, because national ILI reports are much more reliable than data collected from local agencies.

**Fig 2:**
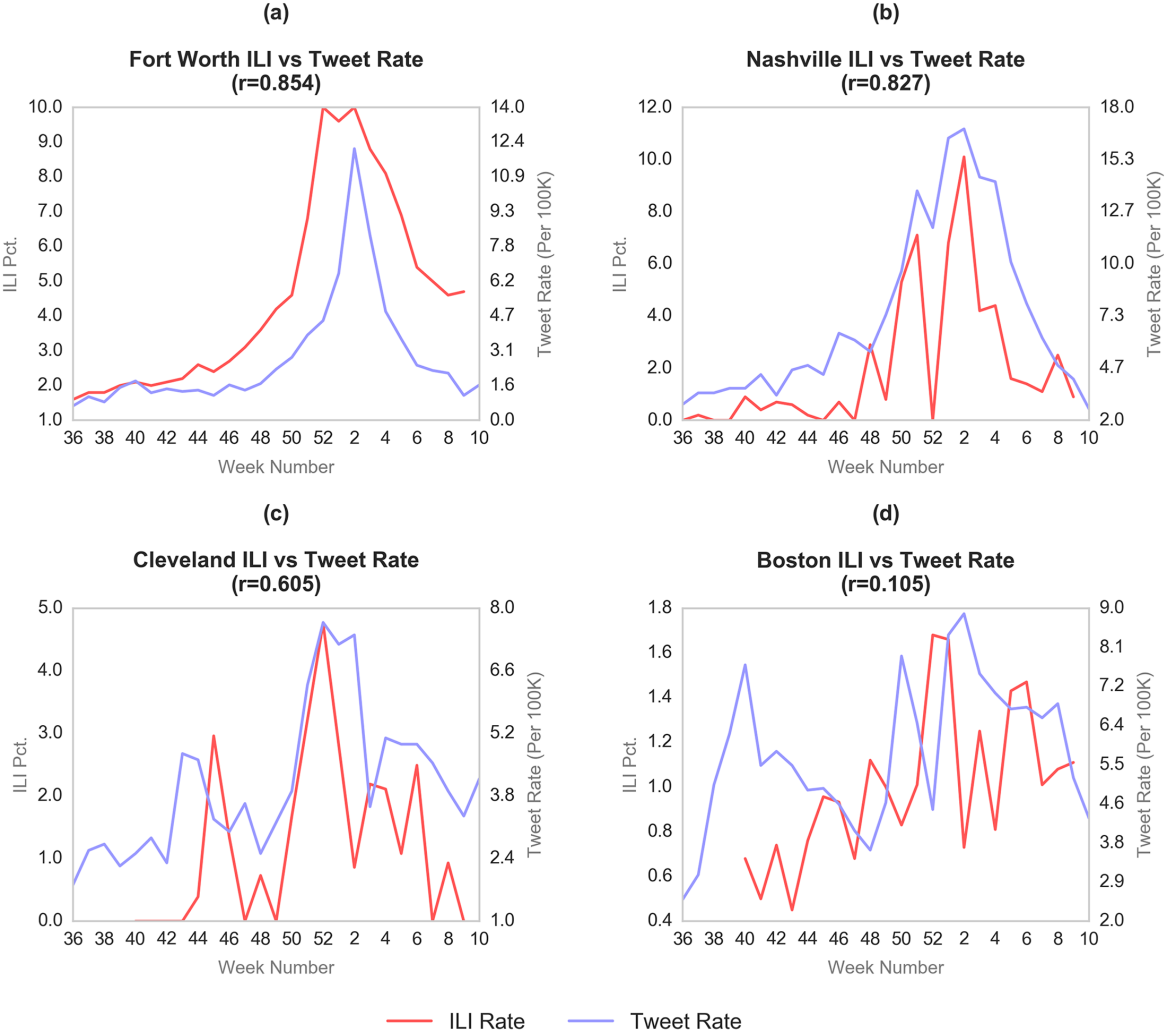
Local sentinel-provided ILI compared to the tweet rate for Fort Worth (a), Nashville (b), Cleveland (c), and Boston (d).

Comparisons between local ILI percentages and tweet rates for individual cities had mixed results, but many of the coefficients were significant. For instance, Fig 2a shows that Fort Worth had a coefficient of 0.854. Similarly, the Nashville-Davidson region (Fig 2b) demonstrates a close relationship between flu-related tweets and local ILI reports (r = 0.827), despite an apparent gap in ILI data for week 52.

A number of cities produced results that were less satisfactory. In certain cases, this may be due to unreliable reporting of local ILI. As shown in Fig 2c, the coefficient for Cleveland is not as high as other cities; however, the curve for local ILI (in red) is very jagged, often dropping abruptly to 0.0%, which suggests that ILI is not reported reliably in this area. Nonetheless, other cities such as Boston (Fig 2d) appear to have a more consistent ILI curve but still reveal little correlation between tweet rate and local ILI, suggesting that the current filtering and classification methods may require fine-tuning to account for spatial variability.

**Fig 3:**
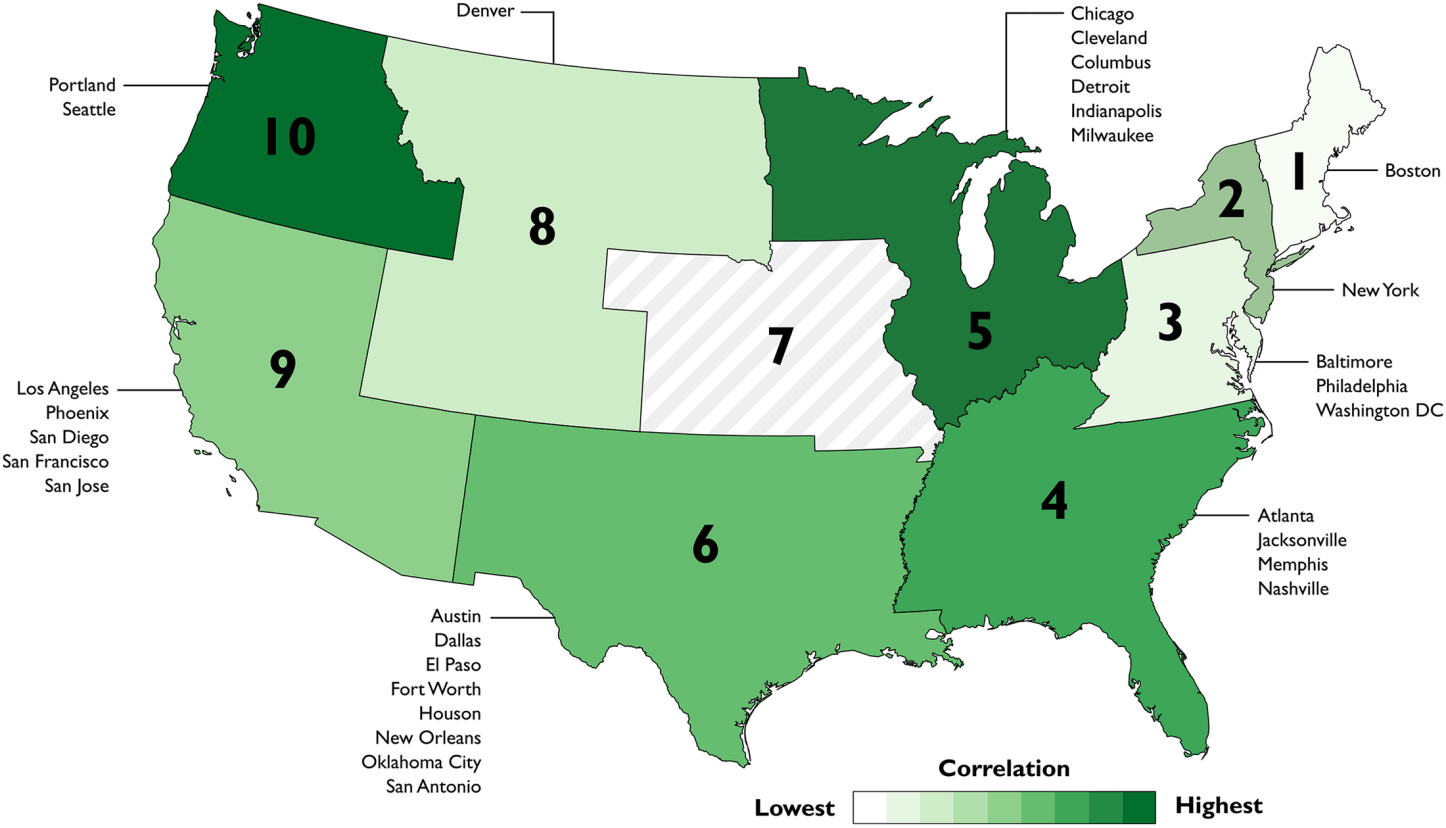
Map showing the correlation rank for each region.

Since local ILI reporting can often be problematic, this study also analyzed twitter activity by taking advantage of regional ILI reports that are made available by the CDC. Table 2 shows each region (and the cities that are contained within) as well as the coefficients between the aggregated tweet rates and the ILI percentages for that region. In Fig 3, the regional correlation results have been mapped to show the geographic variation in the performance of our methods. Unsurprisingly, most regions consisting primarily of cities that showed poor correlations with national or local ILI reports also did not perform well at the regional level. However, aggregating the Twitter data into regions seemed to produce exceptional results in a number of cases. For instance, the correlation coefficient for Region 10 was significantly better (r = .927) than the results for the individual cities of Portland and Seattle. This finding suggests that regional ILI may be used to better evaluate the data collection and filtering methods, as it appears more reliable than local ILI reports, but still accounts for regional differences in the spread of influenza.

**Table 2:** Correlation coefficients aggregated by region. Regional ILI data provided by CDC.

REGION	CORRELATION WITH REGIONAL ILI
Region 1 (Boston)	0.445283886
Region 2 (New York)	0.643321552
Region 3 (Baltimore, Philadelphia, Washington DC)	0.503859481
Region 4 (Atlanta, Jacksonville, Memphis, Nashville)	0.899332773
Region 5 (Chicago, Columbus, Cleveland, Detroit, Indianapolis, Milwaukee)	0.903099689
Region 6 (Austin, Dallas, El Paso, Fort Worth, Houston, Oklahoma City, New Orleans, San Antonio)	0.891701735
Region 7 (No data)	NA
Region 8 (Denver)	0.541016527
Region 9 (Los Angeles, Phoenix, San Diego, San Francisco, San Jose)	0.887259347
Region 10 (Portland, Seattle)	0.927950078

## Limitations

Though this study improves upon previous attempts to analyze the relationship between flu-related tweets and real-world outbreaks of influenza, there are remaining issues that might be addressed in future work.

Currently the support vector classifier used to filter tweets was trained with data from the 2012–2013 season. While this training set produced satisfactory results with respect to the filtering process, this approach to classification ignores the dynamic nature of social media data. For instance, our team discovered that the signal for flu-related tweets in Baltimore for the 2013–2014 season were anomalous in that they did not closely correspond to local ILI reports. After manually analyzing individual tweets for the city, we identified many messages in Baltimore mentioning the phrase “fresher’s flu”, which is a colloquial term to describe illness that sometimes accompanies the start of a college semester. Though these tweets may be indicators of real-world illness, the term “fresher’s flu” generally does not refer to actual influenza. After adjusting our filtering process to account for this anomaly, the correlation coefficients for Baltimore were similar to other cities. This incident demonstrates the need to develop a framework for continually re-training the tweet classifier so that it can account for the temporal and spatial dynamics of messages in cyberspace.

Additionally, the regional analysis of flu tweets presented in this paper might be further improved by including a greater number of search areas. Since Twitter messages were only collected for 31 major U.S. cities, regions with few large urban areas contained sparse data. Three regions (1, 2, and 8) contained tweets from only one city and one region (7) was not covered by any of our search areas. Including more search areas to cover less densely populated areas could address this issue.

Finally, it should be noted that if social media is used to systematically monitor influenza in the future, there are potential concerns that may need to be considered in order to maintain the reliability of this method. For instance, public awareness of these methods could influence behavior and consequently lead to false reporting. It is not hard to imagine a scenario where Twitter users would falsify flu-related tweets in order to garner more attention from public health officials and receive more resources such as vaccination supplies. However, as discussed by Petróczi and Haugen [15], such false reporting may be counterbalanced by further understanding the motivations individuals may have for distorting the truth, and these insights may allow researchers to identify Twitter messages that are likely to be insincere.

## Conclusions

This study demonstrates that social media holds great potential for monitoring the outbreak of flu and other illness. It builds on previous work but suggests that GIS methods can augment existing approaches. Additionally, this paper introduces a machine learning procedure to filter out noise from collected tweets—a task that has been a long-standing hurdle preventing researchers from taking Twitter seriously as a source of data. The outcome demonstrates that this procedure yields results that correlate more strongly with national and local ILI reports.

## Supporting Information

S1 File A Microsoft Excel file containing tables that list influenza-like illness (ILI) rates at local, regional, and national levels. (XLSX)

S2 File A Microsoft Excel file containing tables that list flu-related tweet counts at local, regional, and national levels. (XLSX)

S3 File A document containing links to example source code for collecting geo-targeted Twitter data and the web interface that is used to view and download Twitter data. (DOCX)
