# Supplementary material for: Applying GIS and Machine Learning Methods to Twitter Data for Multiscale Surveillance of Influenza
Source: PLoS One. 2016 Jul 25;11(7):e0157734. doi: 10.1371/journal.pone.0157734 (PMC4959719; doi:10.1371/journal.pone.0157734)
Supplement: S3 File — (DOCX) [file pone.0157734.s003.docx]

**LINKS TO THE TOOLS DISCUSSED IN THE PAPER**

The dashboard we used to visualize social media data related to influenza is at the following URL:

<http://vision.sdsu.edu/hdma/smart/>

An example of a script that can be used to collect Twitter data can be found on our GitHub account:

<https://github.com/HDMA-SDSU/HDMA-SocialMediaAPI/blob/dev/API-Twitter/Twitter_searchAPI_DEMO.py>
